# The Management of Spina Bifida in Bristol

**Published:** 1969-01

**Authors:** J. B. M. Roberts


### THE MANAGEMENT OF SPINA BIFIDA IN BRISTOL

BY

J. B. M. ROBERTS, F.R.C.S.

Treatment of children born with the major congenital anomaly of spina bifida is becoming an increasing problem within the United Kingdom and more than ten years ago it was suggested that this condition was replacing tuberculosis as a main entity in our residential schools for the physically handicapped. Because of the success of early treatment it seems inevitable that the total number of cases to be cared for will grow steadily for several years before the figure finally becomes stabilised. From the available statistics it appears that in England and Wales approximately 2,500 children are born alive with this condition each year. In the Bristol Clinical Area it has become apparent that arrangements must be made for the treatment of approximately 35 new cases each year. Each community must decide what arrangements it will make to meet such demands and the time seems ripe for review of the total problem as it is becoming apparent within Bristol. In order to make this review as complete as possible my own clinical opinions are to be supplemented by the views of a parent of a child born with spina bifida.

Whilst very significant advances have been made in many centres in the clinical management of these children it is becoming increasingly apparent that the necessary administrative changes to cope with the situation are lagging far behind the clinical advances, and at the moment there can be little doubt that the major problem is administrative.

As the borderline of therapeutic endeavour advances, society is increasingly faced with ethical problems which recently have been most apparent in the discussions on transplantation of human organs. An equally considerable problem is posed during the decision whether or not to treat children with spina bifida cystica, the majority of whom would inevitably die without treatment in the early days or weeks of life. Once the decision has however been made to commence treatment, it is important that it should be possible to complete the treatment adequately, and at the moment this is not the state of affairs in Bristol.

### The Total Clinical Problem

Spina bifida is probably the most severe of the common congenital abnormalities compatible with life, and it is the result of failure of fusion of the vertebral arches with or without herniation and dysplasia of the spinal cord and its associated membranes. A defect in the vertebral arches is extremely common, being demonstrable by radiology in probably at least a third of the population. Such a state is known as spina bifida occulta and is usually of no clinical significance. When however there is herniation of the contents of the spinal canal as seen in a pure meningocoele in which herniation is limited to the meninges; or when as in a menginomyelocoele, the spinal cord participates in the lesion as well; the patient is faced with an immediate and late risk to life. Unfortunately meningocoele without any obvious neurological lesion is relatively uncommon, and constitutes only 5% of the total cases of spina bifida cystica.

The management of these patients may be divided into measures directed at the primary lesion or the complications. These secondary complications involve several systems (a) the nervous system, (b) the urinary system, (c) the skeletal system and (d) the ano-rectal mechanism.

*The primary lesion*

The child is born with either meninges or spinal cord tissues exposed to the atmosphere, and if no treatment is undertaken the combination of infection, trauma, and drying of the tissues usually results in a fulminating meningitis which is the common cause of death. There is an increasing amount of evidence that if this is to be prevented, surgical closure of the primary defect must be undertaken as a matter of urgency, preferably within the first twenty-four hours of life, and possibly even within the first six hours of life. At this stage it is usually a relatively simple technical procedure, and major plastic surgery only becomes necessary when the lesion is left for several months before repair. The essential basis of the operation is an excision of the redundant membrane followed by closure of the dura and the skin over the cord. There is much evidence that early closure will limit the later neurological defect, although not all are agreed upon this, and there have been advocates of more conservative treatment.

*The secondary complications*

(a) *In the central nervous system*

Because of involvement of neurological tissue in the primary lesion, the majority of these children are born with variable paralysis of the muscles innervated by the spinal cord at and below the level of the lesion. This paralysis is at present irreversible and therapy has to be aimed at utilisation to the maximum of any functioning nervous tissue and innervated muscles. However, there is also a further matter that is susceptible to treatment. This is the high incidence of secondary hydrocephalus associated with spina bifida cystica, caused by an associated malformation of the medulla oblongata within the cervical canal—the Arnold Chiari malformation. This produces obstruction to the exit foramina of the fourth ventricle with ensuing dilation of the lateral ventricles, and consequent hydrocephalus. Untreated, this can lead to a quite grossly deformed head of such a size that the patient is incapable of any movements of the head. Children were formerly seen with such heads, often complicated by gross pressure sores because of lack of movement. It is now believed that early drainage of the ventricle can prevent much of this damage, and, providing secondary pressure atrophy within the brain is prevented, it would appear that the lesion is compatible with a normal level of intelligence. As more than 80% of children born with spina bifida cystica are exposed to the complication of hydrocephalus, it will be seen that an initial operation to close the spinal lesion will in the large majority then be followed by at least one drainage procedure (and possibly more than one) to control the hydrocephalus; and this is but the beginning of the surgical marathon which is the consequence of deciding to treat a child with such a lesion. The commonest procedure at present to control the hydrocephalus involves the insertion of a uni-directional valve into a conduit from the lateral ventricles to the blood stream, usually in the right atrium. Introduction of such a valve of foreign material is associated with a risk of bacterial colonisation, with a consequent recurrent bacteriaemia which cannot be eradicated until the valve is removed, and replaced when the blood stream has been sterilised.

(b) *Urinary system*

The paralysis produced by the primary spinal lesion does not affect only the skeletal muscles but also the bladder and the rectum; and as far as the urinary tract is concerned successful therapy in the majority of cases is only relative and rarely absolute. The neurological defect produces dysfunction of the bladder which in turn leads to either incontinence, obstruction, or a combination of both. Obstruction in the bladder almost inevitably leads to infection, and the two together tend to produce vesico-ureteric reflux with a secondary pyelonephritis which will eventually kill the child if it is untreated. Once the primary neurosurgical operations have been carried out, the major hazard to life lies in the urinary tract, and treatment is aimed at preservation of intact renal function, if necessary to the extent of sacrificing the bladder and establishing some form of conduit. It has been our experience that in children with a meningomyelocoele and any degree of bladder involvement infection is inevitable before the age of two in the girls, and also in the majority of the boys. Such infection frequently fails to respond to either intermittent or sustained antibiotic therapy because of the presence of residual urine within the bladder or because of associated congenital anomalies. It must be remembered that the general principle of the multiplicity of congenital anomalies holds true in spina bifida, and approximately a quarter of these children will be found to have some structural anomaly within the urinary tract as well as the secondary neurogenic lesions. Thus for the preservation of life the child will probably have to undergo, as well as primary closure of the back and drainage of the hydrocephalus, the establishment of an ileal or colonic conduit to allow free drainage of the kidneys. All these major procedures are usually required within the first five years of life and can only be regarded as life-saving procedures. Apart from the primary closure of the back they do little to advance the state of the child towards independence and a reasonable place in society.

(c) *Orthopaedic complications*

It is however the orthopaedic surgeon who can do most to improve the lot of these children. The variable paralysis leads to muscle imbalance about joints with subsequent deformities if it is not corrected at a sufficiently early stage. The transposition of functioning muscles to other points about a joint in order to mimic the action of those muscles that are paralysed has resulted in approximately a third of the children being able to walk without calipers and a further third who will walk with the aid of callipers. This leaves a minority who will be limited to an invalid chair. It is the realisation that these children can become self-sufficient as far as walking is concerned that has been the most dramatic recent spur to activity. The possibility of employment of a patient with normal intelligence who is capable of existence outside a wheelchair is obviously much higher than in a non-ambulant patient.

(d) *Ano-rectal dysfunction*

As spina bifida most often occurs in the lumbo-sacral region the innervation of the anal canal as well as of the bladder is disrupted. This produces an insensitive anal canal and severe constipation. Anal incontinence is not usu[illegible]

apart from transient episodes of diarrhoea due to constipation. Management of this constipation is entirely conservative and colostomy is not required. A regular mild aperient together with occasional enemata (and in some children manual removal of the dry, hard faeces) usually minimises the problem. Most parents can cope with this situation and rarely regard it as a serious problem. Rectal prolapse is seen in a modest proportion of these children and does not require surgical intervention; its management is not different from that of a similar lesion in a normal child of comparable years, and it will similarly resolve spontaneously in time.

### Social and Psychiatric Consequences

The birth of a child with spina bifida produces a considerable impact on the family unit and there is a great need for intelligent support by both doctors and social workers in the early stages. Time must be available to explain the full implications of this situation, and to reassure the parents that help will be available. Clinicians in this field frequently observe a deep-seated sense of guilt on the part of the parents who cannot accept that the state of their child is other than retribution for past inadequacies on their own part, and they tend at times to become excessively conscientious about the care of the invalid child to the neglect of the other children in the family. Thus the family as a whole, as well as the patient, requires support. If treatment is successful the first five years of the child's life are likely to be dominated by visits to hospital to see various specialists, and assistance must be given to the parents if their life is not to become a constant repetition of visits to hospital with no time for any of the other responsibilities within the family.

### Administrative Problems

As will be appreciated from this clinical introduction, the management of a child with spina bifida necessitates the co-operation of the paediatrician, neurosurgeon, urological surgeon, and orthopaedic surgeon, together possibly with the psychiatrist, the social worker, and others. At the moment in Bristol it is impossible to organise such co-operation as each department functions in a different hospital. Thus parents may well be requested to bring a child to Frenchay for a neurosurgical opinion, to Southmead for a urological opinion, and to the Children's Hospital for a paediatric opinion, all within the space of a few days. Inevitably this results in duplication of sets of notes and inadequate information being available at any one time, and it seems inevitable that if any sort of efficient service is to be produced there should at least be a single out-patient department available. In Sheffield, for example, it has been the practice for the last ten or twelve years to have a combined clinic of all clinicians involved, each using a separate consulting room in the same department. It may well be that ideally a common in-patient unit should also be available. An average patient may well be exposed to ten or more major operations during the first five years of life, and the disruption caused by removal from home life on all these occasions can well be imagined. When however it is realised that instead of going to one hospital unit with which they are familiar they are expected to visit five or more hospitals in the Bristol area, the resulting demands on the child are quite obviously grossly excessive.

If the management of children with spina bifida is to be undertaken at all within this city it is essential that there should be adequate facilities available to allow the treatment to be carried out without imposing a superhuman load on a family that is already facing a problem which many of us would consider severe enough in itself.

### A Parental Viewpoint

" When our child was born we were told she had a ' split in the spinal column ', that she might live but would never walk. No reason was put forward why this should happen to us, particularly in view of our two previous and totally normal children. We were left with no very clear idea of the meaning or the implications of a spina bifida baby, and certainly the enormity of our future tasks as parents of one of these unfortunate children was never honestly broached.

We realise that a general practitioner does not see many of these babies, and as a result is not sufficiently informed to act as a true guide and mentor. We feel therefore that before the child is discharged from the hospital to home, *someone* should discuss very fully the future possibilities and management of the child with the parents.

During the three years of our child's life we had to attend numerous out-patient clinics at different hospitals in the Bristol area— each hospital having a different set of notes—and we received differing opinions. When our child had to be admitted for various procedures, there was only one hospital that seemed to provide nearly ideal surroundings, and in particular we would mention the sister in charge of the ward. All the children in the ward were happy, and the co-operation between this sister and the medical staff, and their relationship with the parents, was superb.

Our whole family life had to be altered to centre around our invalid child. We were worried about the reaction of our other children, but mercifully this proved unfounded as they accepted her as part of the family and she was included in most of their activities.

We do feel that the Medical Social Services could produce more tangible and co-ordinated support, especially when there are other young children in the family. To arrange sometimes three hospital visits per week, and to care for one's other young children, is very difficult. The Health Visitor paid duty visits but offered no real assistance—in fact we found that no-one outside the hospital was really interested. It would seem to us that there is a great need for a base hospital, ideally on the lines of ' A ' Block at Ham Green Hospital, which was the only place that seemed orientated to cope with our child and ourselves.

As it happened our child eventually succumbed. We still miss her and want her, but in retrospect I think perhaps it is asking too much of parents to undertake the day-to-day care of a spina bifida child with the prospect of losing it after a few years. One's hopes are raised, and dashed, and had she not been the subject of quite so many ' life-saving ' procedures in her early days of life, and had she quietly been allowed to take her natural chance of survival without surgical intervention, we might not have missed her quite so much.

There is no replacement for our daughter and for this reason we could not entertain a further pregnancy to compensate us for her loss, let alone take the risk of another similar experience, although this opinion may not be shared by all the parents of a spina bifida child."

